# Supplementary material for: Pharmacokinetics, pharmacodynamics, and toxicity of a PD-1-targeted IL-15 in cynomolgus monkeys
Source: PLoS One. 2024 Feb 5;19(2):e0298240. doi: 10.1371/journal.pone.0298240 (PMC10843171; doi:10.1371/journal.pone.0298240)
Supplement: Attachment — Submitted filename: Responses to Reviewers_Final.docx [file pone.0298240.s002.docx]

PONE-D-23-18791

Pharmacokinetics, pharmacodynamics, and toxicity of a PD-1-targeted IL-15 in cynomolgus monkeys

PLOS ONE

5. Review Comments to the Author

Please use the space provided to explain your answers to the questions above. You may also include additional comments for the author, including concerns about dual publication, research ethics, or publication ethics. (Please upload your review as an attachment if it exceeds 20,000 characters)

**Reviewer #1**: General Comments

This is an interesting and potentially important paper from an industry team, which clearly represents substantial work. The literature context is nicely described, and the interpretation of much of the data is reasonable. However, more detail is needed in several areas.

Specific Comments

Animal care, human criteria for euthanasia, and euthanasia drugs/details should be provided. The authors should apply the ARRIVE guidelines for reporting of animal experiments.
Author Response: we have added some additional information to these monkey studies.

The basis for the numbers of animals used should be stated. Statistical justification may not be possible with such a low N, but a rationale or guideline could be given.

Author Response: The number of monkeys used in the IND-enabling GLP tox study was following regulatory guidelines (ICH S9). We added a sentence on Page 8 under “Cynomolgus monkey GLP toxicity study”.

The data availability statement is not acceptable; specific details are required. The authors are encouraged to read the instructions.

Author Response: Animal study related information are stored at Pfizer database and additional information beyond those in the manuscript can be provided upon request to the corresponding author.

Lines 6 and 7: Abbreviations should be spelled out.

Author Response: they were spelled out.

Line 31: The range for T1/2 is broad, 8-13 hours; suggest providing a mean.

Author Response: We modified the sentence to: “PF-07209960 showed dose dependent pharmacokinetics with a terminal T1/2 of 8 and 13 hours following IV administration at 0.03 and 0.1 mg/kg, respectively. The clearance is faster than a typical IgG1 antibody”

Lines 99-123 and elsewhere in the methods: Please define ambiguous abbreviations on first use; some are defined later in the paper or not at all.

Author Response: Thanks for this suggestion, we added the definitions of these abbreviations.

Lines 125-132, 178-179, Tables, Figures: More details are needed regarding the animals.  
How many animals were used? There are different numbers in different parts of the paper, with some information emerging only in the figure legends. Please clarify total number of males and females and age of animals used in each study up front in the methods.

Author Response: Thanks reviewer for this suggestion. In this manuscript, data from two separate monkey studies are used: PKPD study and the GLP tox study. We added the number of animals and other information to these two studies in the M&M section.

What was the genetic strain and provenance of the animals?
Were the animals treatment naïve? If they were returned to the colony after this study, does that mean they might have been in prior studies of antibody-like drugs? If so they might have pre-existing ADA to similar agents.

Author Response: All monkeys used in this study are naïve monkeys. We also added the monkey origin, BW, and age information to the PKPD study and GLP tox study sections in M&M.

Lines 171-175: Please use past tense if the work was actually conducted as described. Please remove the use of infinitive or future tense.

Author Response: Thanks for pointing this out. We made the appropriate changes.

Lines 176-177: The statement “(that do not require analyses)” is unclear, please explain or remove.

Author Response: Good point, we deleted it.

Lines 180-183: Please describe the frequency of animal health assessments.

Author Response: we Added these information.

Lines 183-184: Please describe the timing and frequency of blood collections for PK and ADA analysis.

Author Response: We added the timepoints.

Line 186: Please provide the dose of pentobarbital used.

Author Response: 50 mg/kg, added this to the updated manuscript.

Line 188: “may include” is vague, please just give the criteria that were actually used.

Author Response: We made the modification. Deleted “may”. These are the general guidelines of unscheduled euthanasia. Under which, we also described the specific reasons for the authorization of the animals in our study.

Lines 196-198 and 392-404: Please provide a list of organs that were were examined. Were any tissues unaffected? Were there differences by dose? What were the findings in animals that did not survive until day 31? Were all animals affected identically? A table would be helpful.

Author Response: The list of all organs/tissues examined and the microscopic examination findings (unscheduled and scheduled) in the GLP toxicity study have been added as supplemental tables. We also added more detailed information about the unscheduled euthanasia in the Result section. In general, histological findings are similar in these unscheduled euthanasia animals, however, since the mortality is caused by cytokine release, individual animal differences in tissue histological change presentations are expected.

Line 217: How long were cells refrigerated before flow cytometry analysis? Please specify at least an upper limit, “less than ___”.

Author Response: We added this information (less than 72 hours) in the updated manuscript.

Line 234, 285 and elsewhere: Please correct the document for errors in English grammar, for example, the appropriate use of articles “a” “an” “the”, etc.

Author Response: Thanks the reviewer for pointing these out. Corrections have been made in the updated version.

Table 1: Footnotes are excessive; please put the information about sex and time in the body of the table. Author Response: Thanks for the suggestion. We can removed the AUC time footnotes, since the column title is AUCt and we don’t have to specify the time for the last measurable concentration in each dose group in the table.    We leave the animal # and sex in footnote.

Line 320-321: This sentence needs clarification. Does 6 hours refer to the SQ route?

Author Response: Thanks the reviewer for identifying this error. We made the correction below: “Following each dosing, nearly 100% of cell surface PD-1 were engaged by PF-07209960 within 15 minutes (IV route) or 6 hours (SC route) at all dose levels, respectively.”

Line 375-391: A statement of fold change does not provide enough information. For example, an 11-fold increase in neutrophil count is noteworthy but it is not possible to interpret without knowing “11 fold of what”; similarly for platelets and clinical chemistry. Please provide absolute and differential counts.

Author Response: This section has been updated to include the most extreme absolute value for key parameters

Line 384: Were the animals septic?

Author Response: No, none of the animals were septic.

Figure 1E: Please specify the day of measurement.

Author Response: measured on Day 1 predose, added this information to the figure legend.

Line 533: How many animals underwent unscheduled euthanasia for illness in the 1 mg/kg group?

Author Response: 4 in total.

Figure 5: The authors suggest that thrombocytopenia was a transient sequestration effect; this might be true at the low doses, but why was there such profound thrombocytopenia in the animals that succumbed? Was there possibly a consumptive coagulopathy, or an anti-platelet effect of the drug? Please show data from all groups.

Author Response:

Graphs have been updated to include data from all groups.

There was evidence of activation of the coagulation cascade in some of the animals that underwent unscheduled euthanasia, as indicated by prolongation of prothrombin time (PT) and activated partial thromboplastin time (APTT); however, prolongations were not confined to or notably more severe in the individual animals with the most profound thrombocytopenia. Additionally, there were consistent increases rather than decreases in fibrinogen, and there was no microscopic evidence of disseminated intravascular coagulation. Further complicating clear identification of coagulation cascade activation in these animals is the fact that false APTT prolongations can occur in inflammatory conditions (ref below). However, it is certainly possible there was an additional or alternate effect other than sequestration on platelets, especially in unscheduled euthanasia animals; the Discussion section has been updated to reflect this.

*(van Rossum AP, Vlasveld LT, van den Hoven LJ, de Wit CW, Castel A. False prolongation of the activated partial thromboplastin time (aPTT) in inflammatory patients: interference of C-reactive protein. Br J Haematol. 2012 May;157(3):394-5.)*

**Reviewer #2**: The IL-15-PD-1 molecule produced by the investigators is exciting and important for the field. It represents a shift in drug development to bispecific molecules. The current study is a follow-up from a prior preclinical study.

Similar to other IL-15 agents, the study drug induced expansion and KI67 of lymphocyte subsets in the periphery. Do these lymphocyte subsets express PD-1? If not, is the study drug acting in a PD-1 dependent manner? Is it possible that reduced dosing would target PD-1hi cells without PD1neg lymphocytes? _

Author Response: This is a great comment. Unfortunately, we didn’t measure PD1 expression or PD1+ cell % change kinetics in the GLP tox study. We have shown in our previous publication that our drug has higher activity on T cells that express PD1 (*Xu Y, et al. An Engineered IL15 Cytokine Mutein Fused to an Anti-PD1 Improves Intratumoral T-cell Function and Antitumor Immunity. Cancer Immunol Res 2021;9(10):1141-57*). We also think that by reducing dose, we probably can see lower activity on PD1- T cells in peripheral blood or tissues while maintain sufficient activity on tumor infiltrating T cells that express higher PD1.

Figure 3 reports fold change. Most studies of this nature show the percentage of the marker under evaluation or the percentage of a particular lymphocyte population. Providing data graphed in this manner would be helpful for comparison purposes. Also, can the authors provide their data on NK cell KI67 expression? This seems relevant given the authors argument that NK cells will not be targeted as efficiently.

Author Response: Unfortunately, we didn’t analyze Ki67+ NK cells in this study. For the lymphocyte subsets, the effect on them by PF-07209960 is expansion/proliferation, and the best way of showing this is the change of cell numbers (counts) from baseline values (fold change). There is little if any effects on the percentages of these subsets. We agree with the reviewer that adding graphs of the percentages of Ki-67 and CD69 positive T cells can provide additional information. We added these graphs to Fig 3.

The authors compare their drug to other monkey studies with IL-15. It may be useful to compare results with other IL-15 studies where IL-15 is linked to an Fc. Rhode et al report in Cancer Immunology Research in 2016 “Comparison of the super agonist complex, ALT-803, to IL-15-“

Author Response: This is a great question. Since this is a drug development program and we need to be focused on our compound PF-07209960 and generate minimal in vivo toxicity study data that meet regulatory requirement. Therefore we didn’t include competitor’s IL-15 molecules in our toxicity studies. However, our data is generally consistent with Rhode’s publication with some differences (eg. PF-07209960 showed less potent proliferative effect on NK cells compared with T cells, while ALT-803 has the opposite effect, more potent pro-proliferative effect on NK cells than on T cells).
 
Is there prior literature about platelets and high dose IL-2 that may be useful for the authors observations? Oleksowicz – Dutcher in 1994 may be useful.

Author Response: Although the effects we saw were unique in some ways from the effects described in the literature in association with IL-2 administration, it is possible there were other causes/contributors to the platelet decreases observed in this study. The Discussion section has been updated.

The authors write in the methods that samples were refrigerated until flow cytometry analysis. What is the range and the maximal time before flow cytometry?

Author Response: less than 72 hours before being analyzed on a flow cytometer. We added this information to the Fig legend.
